# Do self-reported stress and depressive symptoms effect endothelial function in healthy youth? The LOOK longitudinal study

**DOI:** 10.1371/journal.pone.0196137

**Published:** 2018-04-23

**Authors:** Lisa S. Olive, Walter P. Abhayaratna, Don Byrne, Alice Richardson, Richard D. Telford

**Affiliations:** 1 School of Psychology, Deakin University, Melbourne, Victoria, Australia; 2 ANU Medical School, Australian National University, Canberra, Australian Capital Territory, Australia; 3 ANU College of Health and Medicine, Australian National University, Canberra, Australian Capital Territory, Australia; 4 Research Institute of Sport and Exercise, University of Canberra, Canberra, Australian Capital Territory, Australia; Stellenbosch University, SOUTH AFRICA

## Abstract

**Background and aims:**

Endothelial dysfunction is thought to be an early indicator of risk for cardiovascular disease and has been associated with both stress and depression in adults and adolescents. Less is known of these relationships in younger populations, where the origins of CVD is thought to manifest. This study examined the effects of questionnaire derived psychosocial stress and depressive symptoms on endothelial function among children, following them through to adolescence.

**Method:**

Participants were 203 grade 2 children (111 girls; M age = 7.6 ± 0.3 years) from the LOOK longitudinal study, who were followed through to adolescence (16 years). Self-reported psychosocial stress and depression were assessed using the validated Children’s Stress Questionnaire and a modified and validated version of the Children’s Depression Inventory respectively; endothelial function was assessed using EndoPAT 2000 system at follow-up only; and adjustments were made for fitness, pubertal development and socioeconomic status.

**Results:**

Although all relationships occurred in the hypothesised direction, no cross-sectional or prospective evidence of early symptoms of psychological stress or depression being associated with endothelial dysfunction was found among our asymptomatic cohort of adolescents (all *p* > .05).

**Conclusions:**

In contrast to previous findings in adolescents, our data provided little evidence of any relationship between current or previous psychosocial stress or depression and endothelial function in 16-year-old boys and girls. However, our data need to be interpreted alongside the potential limitations in the sensitivity associated with self-report methods for detecting psychological distress of children.

## Introduction

Chronic life stress and depression have been identified as significant risk factors for the development and prognosis of cardiovascular disease (CVD) [1–3]. There is also evidence that children experience stressors both in the form of ongoing daily hassles [4] and stressful life events [5]. In addition, a proportion of children will experience either clinical or subclinical levels of depression, with estimates for the prevalence of diagnosable depressive disorders in children and adolescents ranging from 3% to 6% [6, 7]. While CVD generally manifests in adulthood, the pathological processes leading to CVD may begin much earlier [8–11], and given these data on childhood mental health, there is the potential that psychological distress experienced early in life may contribute to early vascular dysfunction. Advances in non-invasive assessments of vascular function, a predictor of CVD [12], have opened up research possibilities with younger populations to examine the effect of early psychological distress on vascular function and future CVD risk. While the precise mechanisms that may underlie relationships between psychological characteristics and risk of CVD are not well understood, endothelial dysfunction is one potential mechanism that may link early stress and depression with increased risk for CVD [13].

The vascular endothelium plays a crucial role in maintaining vascular homeostasis and regulates several physiological functions, including vascular tone and vasomotor function. Endothelial dysfunction, which is described as “an imbalance between the vasodilating and vasoconstricting milieu of the endothelium” [14], has been linked with the pathobiological processes of vascular inflammation, platelet aggregation and thrombosis [15]. Endothelial dysfunction has previously been shown to occur in healthy, asymptomatic children with no clinical evidence of vascular disease [11], supporting the notion that endothelial dysfunction may be one of the earliest stages in vascular damage and an early maker of future CVD risk.

Among adults, there is evidence to suggest that depressed mood is inversely correlated with endothelial function in both healthy CVD-symptom free adults and individuals with CVD or at risk of CVD [13, 16, 17]. Similar to depression, brief episodes of psychosocial stress have been shown to affect endothelial dysfunction in a number of experimental and observational studies of adults [18, 19]. More recently, an emerging literature is beginning to document the adverse impact of depressive symptoms and mental stressors on endothelial function in adolescents and younger populations, in both clinical samples and those drawn from the general population, with no known psychopathology [20–24]. While this area of research is relatively novel in younger populations, it is hypothesised that these psychological effects do not suddenly emerge as risks to CVD upon reaching adulthood; that rather psychological distress may induce the early stages of the development of CVD in children and adolescents, with one potential mediating pathway being impaired endothelial function.

Longitudinal cohort studies are ideal for investigating how patterns of psychological health change over time and relate to changing exposure patterns and the development of CVD. On the other hand, longitudinal studies are costly to instigate and maintain, which often limits the types of assessments that can be undertaken. To date, few prospective investigations on indicators of psychological disturbance and endothelial function have been undertaken with younger, asymptomatic populations, limiting our understanding of the origins or the necessary threshold of psychological disturbance as a risk factor for CVD. Yet, preliminary evidence previously discussed indicates that this may occur in childhood/adolescence, even with subclinical levels of psychological disturbance [22, 23]. In the current study, we sought to investigate whether stress and depressive symptoms effect early disturbances in endothelial function among the Lifestyle of our Kids (LOOK) study cohort of asymptomatic children with no known clinical symptoms for CVD, following them into adolescence.

## Method

### Participants

This study forms part of the multidisciplinary Lifestyle of our Kids (LOOK) project [25]. We obtained endothelial data from 203 children (92 boys, 111 girls) recruited from 29 public primary schools in the Australian Capital Territory (ACT), which forms the sample of the current study. Children were sampled from the general population and at the commencement of the study, were apparently free from clinical psychopathology as reported by a parent and with no known clinical symptoms for CVD, as assessed by blood screening and echocardiographic assessment carried out during the LOOK study. Approximately 90% of the LOOK cohort had one or both parents who were of European descent, 7% of Asian descent, 1% was indigenous Australian or Polynesian, and 2% were unknown.

### Measures

Data were collected in grade 2, grade 3, grade 6 and grade 10, with the exception of endothelial function, which with advancements in technology (not being readily available or reliably tested during the earlier measurement periods of the study), was introduced into the LOOK study in grade 10.

#### Psychological assessments

Measures of stress and depressive symptoms were collected at school in class groups. Items were presented to children via a PowerPoint presentation and participants made response choices on individual hand-held key-pads, which were relayed back to a lap-top computer using KEEpad interactive software and devices (LUL Technology). The response device presented facial images representing a corresponding idiographic scale on individual key-pads, colour-coded to match the response colour presented on the PowerPoint slide in order to facilitate self-reports of stress and depression. All items were simultaneously read to participants as they were presented to take account of varying levels of reading ability across classes.

#### Children’s Depression Inventory

Depressive symptoms were measured using a modified version of the Children’s Depression Inventory (CDI) [26]. The CDI has demonstrated validity and reliability in assessing depression in pre-adolescent and adolescent groups [26–28]. Modification to the original scale was necessary to gain acceptance for use in primary schools by a number of school principals and from the jurisdiction’s Education Department. These modifications have been validated and are described in detail elsewhere [4, 29] but briefly, led to the removal of all items indicating conspicuous clinical depression (e.g. persistent crying—item 10, suicidal ideation—item 9, and worthlessness—items 7) because the inclusion of such items may have inadvertently induced an unpleasant and potentially lasting negative mood state in a very largely psychologically normal sample, which was deliberately unselected for either mental or physical dysfunction. The modified CDI comprised 19 items, with response choices limited to two (symptom present or absent). This resulted in a full-scale score of depression that ranged from 19 to 38, with higher scores indicating greater severity of depressive symptoms. Although a clinically significant cut-off point is difficult to determine in the current modified version of the CDI, where item response options were restricted to symptom absent or symptom present, using the same ratio as the clinical-cut off outlined in the original CDI, a cut-off point of 26 would apply in the current research, with scores equal to or higher than this indicating that the child is experiencing a level of symptoms that could be severe enough to be meet a clinical diagnosis for a mood disorder. Of course the use of this cut-off should be treated with caution and does not translate to a clinical measure per-se, but it may provide descriptively useful and therefore should not absolutely preclude the use of the cut-off in characterising the sample.

#### Children’s Stress Questionnaire

Stress was measured using the Children’s Stress Questionnaire (CSQ) [4], which was developed for the LOOK study and based largely on the widely used Adolescent Stress Questionnaire (ASQ) [30], a validated and reliable measure of psychological stress in adolescence. The CSQ is a 50-item self-report inventory assessing the degree of self-reported impact of stressor experience over the past year. Children are asked to rate how stressful they found each event on a 5-point Likert scale (1 = *‘This did not happen to me’*, 2 = ‘*It happened but it didn’t matter to me*’, 3 = ‘*It made me a bit upset’*, 4 = ‘*It made quite ups*et’, 5 = ‘*It made me very upset*’). This resulted in the entire inventory score spanning 50 to 250, with higher scores indicating greater stress.

Both the CDI and the CSQ demonstrated good internal reliability (CDI Cronbach’s Alpha range = .77- .86; CSQ Cronbach’s Alpha range = .90 to .93) over the eight-year period of data collection.

#### Endothelial function

Endothelial function was assessed non-invasively by trained technicians using the EndoPAT 2000 (Itamar). The EndoPAT device captures a beat-to-beat plethysmographic recording of the finger arterial pulse wave amplitude with pneumatic probes. A peripheral arterial tonometry (PAT) probe is attached to the index finger of each hand, one forming the test finger and the other the control. The EndoPAT examination involves three phases: (1) the baseline phase, which is recorded for 5 minutes, (2) the occlusion phase, where a blood pressure cuff is inflated to supra-systolic pressure for 5 minutes on the test arm, and (3) the reactive hyperaemia phase, which occurs after the cuff is released and the signal is recorded for 5 minutes. In a healthy individual with no known CVD-symptoms, pulse amplitude will increase rapidly after cuff deflation, representing increased blood flow, called reactive hyperaemia. An attenuated response after cuff deflation is an indication of impaired endothelial function. Data obtained from the control finger are used to adjust for systemic effects during the computerised algorithm calculation. In the current study, a standardized algorithm proposed by the manufacturer was used to calculate the reactive hyperaemia index (RHI) [31]. EndoPAT has demonstrated reliability and validity as a measure of endothelial function, including preliminary evidence for its use in paediatric populations [32, 33], and has accurately identified the early stages of atherosclerosis [34].

#### Cardiorespiratory fitness

The 20-metre multi-stage shuttle test (MSST) was used as a measure of cardiorespiratory fitness (CRF) and has been well established as a reliable field-test of fitness among children [35]. The MSST requires maximal effort and therefore performance may be influenced by participant motivation, but its reliability was enhanced by its administration by the same exercise scientist across each data collection time-point and therefore limiting variation is assessment approach.

#### Pubertal maturation

The self-report Tanner stages of pubic hair, breast development, and date of menarche were employed [36] using diagrams based on those previously described [37]. The self-assessment was completed at a hospital paediatric unit under the supervision of an experienced teacher. While physical examination by a physician may be preferred to self-report assessments of pubertal maturation, ethically this approach was viewed as an undue intrusion on the privacy of children and not acceptable to the jurisdictions Education Department. Therefore, we chose to use self-report assessments rather than no assessment, which is a common approach in large scale cohort studies.

#### Socioeconomic status

The Australian Bureau of Statistics (ABS) Socio-economic indexes for areas (SEIFA) [38] was used as a measure of socioeconomic status (SES), as previously described [29]. For suburbs in which the children resided, the mean ± SD index was 1085 ± 40 (range, 982–1160), with the mean being higher and the range being lower than those for all towns and cities in Australia (mean ± SD index, 980 ± 84; range, 598–1251).

### Ethics

This study was approved by the ACT Health Department Research Ethics Committee, and by the Human Research Ethics Committees of the Australian National University, the ACT Department of Education, and the Australian Institute of Sport. Parental consent was obtained for all measures in this study, and children understood that their participation was entirely voluntary and that they could withdraw at any time. The children also provided written consent for psychological measures, a condition requested by the Human Research Ethics Committee of the Australian National University.

### Statistical analysis

Hierarchical linear regression was used to assess the prospective effect of psychosocial stress and depression at each time point on endothelial function at age 16 years. In addition, general linear modelling was used to quantify and assess the effects of change in psychosocial stress and depression between grade 2 (7 to 9 years) and grade 10 (16 to 17 years) on endothelial function in grade 10. Children who missed an assessment in any particular year remained in the study and were included in the analysis, with the statistical model adjusting for missing values. Missing data was replaced using multiple imputations (LISREL v8.7).

Our models included and adjusted for the potential confounding effects of gender, SES, CRF, pubertal development and school, taking account of the “cluster” unit of variation in our design. In our preliminary modelling, we first included percent body fat to adjust for potentially confounding effects. However, we found no significant relationships between DEXA derived assessments of percent body fat (all *p* > 0.05) and endothelial function. We chose to remove percent body fat as a confounding factor from our final model due to collinearity concerns with our measure of cardio respiratory fitness (20m multistage shuttle run), which as a function of the assessment itself, adjusts for percent body fat (i.e. a child must carry their body weight during the 20m shuttle run–both lean and fat mass, which effects cardio respiratory performance). General model checking procedures were routinely used to identify aberrant data and to check the model assumptions. Statistical computation was undertaken using the statistical package R version 3.1.1 [39].

## Results

Table 1 shows unadjusted characteristics of the participants at each assessment.

**Table 1:** Unadjusted means (standard deviations in brackets) for measured characteristics of boys (M; N = 92) and girls (F; N = 111) included in our study at grade 2, grade 3, grade 6, and grade 10.

		Grade 2	Grade 3	Grade 6	Grade 10
		*M (SD)*	*M (SD)*	*M (SD)*	*M (SD)*
EndoPAT	*F*	Not obtained	Not obtained	Not obtained	2.12 (0.56)
	*M*	Not obtained	Not obtained	Not obtained	2.20 (0.59)
Psychosocial Stress	*F*	93.48 (20.28)	94.31 (23.91)	83.90 (19.13)	87.87 (18.31)
	*M*	91.93 (23.50)	90.30 (23.75)	80.53 (22.68)	82.84 (20.06)
Depression	*F*	24.90 (3.88)	24.67 (4.63)	22.75 (2.90)	27.56 (4.72)
	*M*	24.98 (4.39)	24.16 (4.26)	22.98 (3.03)	25.91 (4.38)
^a^Tanner Stage	*F*	Not assessed	Not assessed	2.60 (0.78)	4.33 (0.60)
	*M*	Not assessed	Not assessed	2.50 (0.87)	4.32 (0.67)
^b^CRF	*F*	3.47 (1.08)	3.88 (1.28)	5.48 (1.77)	5.86 (1.93)
	*M*	4.15 (1.46)	4.89 (1.83)	6.39 (2.14)	9.01 (2.71)

^a^Tanner stage = self-assessed pubertal stage ranking;

^b^CRF = cardiorespiratory fitness, the number of stages completed in the multistage run

### Endothelial function

While the average EndoPAT score for the current sample was in the healthy range (*M* = 2.21, *SD* = 0.62), with reference values for EndoPAT RHI indices ≤1.67 indicating endothelial dysfunction (Itamar-Medical, 2015), 21% of children recorded RHI scores of 1.67 or below (indicating endothelial dysfunction). A further 19% of participants recorded RHI scores between 1.67 and 2.00, which has been described as borderline endothelial dysfunctional [31]. The remaining (60.1%) participants recorded scores indicative of healthy endothelial function. It should be noted that the EndoPAT RHI index is based on adult data, the clinical significance of which is yet to be determined in younger populations.

### Depression and psychosocial stress

There was an overall increase in depressive symptoms over time, averaging 0.24 points in the CDI per year (*p* <.001). Specifically, this comprised a decrease in depressive symptoms from grade 2 through to grade 6 followed by a significant increase from grade 6 to grade 10 (*p* <.001). While depressive symptoms existed on a continuum of low to severe, based on proposed cut-offs provided by the authors of the original CDI, 32% of children experienced depressive symptoms that might be considered troubling, if not clinically significant at some point in the study. Of course, the use of this cut-off should be treated with caution and does not translate to a clinical measure per-se, but it may provide descriptively useful and therefore should not absolutely preclude the use of the cut-off in characterising the sample.

No significant changes in stress were observed between grade 2 and grade 3 (all *p* >0.05). In boys, psychosocial stress significantly decreased between grade 3 and 6 (*p* = .001), which was followed by a significant increase between grade 6 and grade 10 (*p* <.001). In girls, the only significant change was between grade 6 and grade 10, where stress increased (all *p* <.001) and this was largely due to an increase in stressors relating to school problems. In relation to the types of stressors participants experienced, during elementary (primary) school, the most frequently self-reported stressors among participants related to their relationships with friends (e.g. dealing with friends in a bad mood, arguing with friends and being ignored or teased by their peers). By high school (assessment collected in grade 10, age 15–17 years), participants most frequently reported stressors relating to problems in the school environment (e.g. having to learn things they are not interested in, doing badly on a big test and having too much homework), and ongoing and pervasive daily hassles (e.g. having too many things to do at the one time, having to do things with people they don’t know and having to deal with friends in bad moods) [40].

### Fitness and pubertal development

Effects of pubertal development and fitness were accounted for in our model. There were no significant effects of puberty (boys: *p* = .366; girls: *p* = .309) or fitness (boys: *p* = .897; girls: *p* = .346) on endothelial function at grade 10. However, when utilising data from all years of measurement, a trend for a positive effect of fitness on endothelial function was evident in girls (*p* = .058) but not boys.

### Effect of psychosocial stress and depressive symptoms on endothelial function

A series of hierarchical linear models assessed the effect of both psychosocial stress and depression on endothelial function at each year of measurement. Firstly, we assessed the effect of self-reported depression and stress at each year of measurement (entering all years of data in the same model, with separate models for depression and stress) on endothelial function at grade 10. Although all relationships occurred in the hypothesised direction, there were no significant effects of depression (see Table 2) or psychosocial stress (see Table 3) on endothelial function, either prospectively or concurrently at age 16-years (all *p* >.05). This remained unchanged after adjustment for the potentially confounding effects of cardiorespiratory fitness and pubertal status. Secondly, we assessed whether changes in depression and psychosocial stress between grade 2 and grade 10 were associated with endothelial dysfunction at grade 10, but no evidence emerged to support the hypothesis that children who had an increase in stress and depressive symptoms would also have poorer endothelial function in adolescence (see Table 4). It is worth noting that in our initial modelling, we also investigated relationships between endothelial function with stress and depression separated by gender, and found no significant relationships (all *p* >.05) and a similar pattern of results when compared to our final model using a combined sample.

**Table 2:** Summary of hierarchical regression analysis for depressive symptoms predicting endothelial function at age 16 years.

	*B*	*SE B*	*β*	*p*
*Step 1*				
Sex	0.079	0.128	0.064	.537
CRF	0.023	0.023	0.105	.314
*Step 2*				
Sex	0.077	0.130	0.062	.555
CRF	0.020	0.024	0.091	.414
Depression Grade 2	0.000	0.014	0.003	.973
Depression Grade 3	-0.004	0.014	-0.027	.794
Depression Grade 6	-0.018	0.019	-0.090	.351
Depression Grade 10	-0.001	0.013	-0.010	.919

*R*^*2*^ = 0.008 for Step 1; Δ*R*^*2*^ = -0.26 for step 2 (*p* > 0.05)

**Table 3:** Summary of hierarchical regression analysis for psychosocial stress predicting endothelial function at age 16 years.

	*B*	*SE B*	*β*	*p*
*Step 1*				
Sex	0.079	0.128	0.064	.537
CRF	0.023	0.023	0.105	.314
*Step 2*				
Sex	0.086	0.129	0.070	.506
CRF	0.024	0.024	0.108	.316
Stress Grade 2	-0.001	0.003	-0.054	.596
Stress Grade 3	-0.001	0.003	-0.02	.853
Stress Grade 6	-0.003	0.003	-0.095	.358
Stress Grade 10	0.003	0.003	0.080	.417

*R*^*2*^ = .008 for Step 1; Δ*R*^*2*^ = -.21 for step 2 (*p* > 0.05)

**Table 4:** Longitudinal effects of psychosocial stress and depressive symptoms predicting endothelial function at age 16 years.

	*B*	*SE B*	*β*	*p*
Change Depression 05–13	0.001	0.009	0.012	.883
Change Stress 05–13	0.002	0.002	0.073	.344

Note: Analyses adjusted for sex

## Discussion

This prospective cohort study provided no cross-sectional or prospective evidence of early symptoms of psychosocial stress or depression being associated with endothelial dysfunction among our asymptomatic cohort of adolescents, free from clinical symptoms of CVD and clinical psychopathology. While no effect was apparent in the current cohort, these findings should not be interpreted as in conflict with those in clinical and high-risk populations (for depressive disorder), where significant and negative effects of depression on endothelial function have been found in adolescents [23, 24]. Rather, the current study might best be interpreted in terms of its contribution to the literature concerning healthy children and adolescents, with no known CVD-symptoms or psychopathology. We highlight this distinction as our findings, when taken together with the evidence to date, support that children drawn from the general population, who are in apparently good health (both physically and psychologically) but are still experiencing some stressors (e.g. daily hassles) and some symptoms of depression do not appear to be at risk of impaired endothelial function. That rather, endothelial dysfunction may only begin to occur at more severe levels of psychological distress [24]. Our findings therefore make an important contribution to the field in helping to understand critical cut-offs and critical points in development for when psychological distress may arise as a risk to endothelial function, and later risk for CVD.

In seeking to explain the outcomes of the current study, firstly, participants from the LOOK study were, for the most part, physically and psychologically healthy children, who were not selected on the basis of any psychological disorder or for being at risk for CVD. Indeed, blood screening and echocardiographic assessment carried out during the LOOK study [41, 42], together with documentation from the parents of their child’s wellbeing and ability to participate in vigorous physical activity were good assurances that children were in good mental and physical health. Our self-report assessments of stress and depressive symptoms may be interpreted as early indications of risk for stress- and/or depressive-disorder and therefore, relevant to investigations with endothelial function in terms of prevention and identifying the earliest indicators of risk. Secondly, children of the LOOK study were younger at initial assessment (7 to 8 years), and at the first follow-up (8 to 9 years) than in any previously reported study, including those with asymptomatic youth, where participant mean age ranged from 14 years [43] to 19 years [23] in studies investigating depression, and from 10 to 17 years in the study assessing response to stress [21]. Older children have greater cognitive capacity for interpreting self-report items, so we may expect a more accurate response. Furthermore, older children will have been exposed to more life experiences that potentially impact upon stress and depression, which may in turn expose relationships not apparent in their earlier years. Certainly, within the age range of our cohort, depressive symptoms did increase with age and, adding to the chances of detecting relationships in older participants, endothelial function is known to decline as adolescents approach adulthood [44].

As alluded to above, although our data do not provide evidence that endothelial function is influenced by self-reported symptoms of psychosocial stress and depression in children and adolescents from the general population, this does not preclude such associations being in existence. Plausible biological pathways have been proposed linking psychological distress with endothelial dysfunction, including evidence that both stress and depression induce physiological changes, including dysregulation of the autonomic nervous system and HPA-axis [45, 46]. Stress and depression may also induce behavioural changes, such as changes in physical activity, diet and smoking behaviour [47–49], all of which promote the development and progression of CVD [50]. These physiological and behavioural effects associated with stress and depression give weight to the premise that early psychological factors may influence subsequent cardiovascular health, making the current research warranted, and highlight the need for further research utilising longitudinal designs, which are able to detect change in these associations as youth develop over time. Future studies may also benefit from employing clinician-led assessments of psychopathology (e.g. clinical interviews), should resources permit such a methodology.

Strengths of this study include the relatively large sample size; longitudinal assessments of psychosocial stress and depression with good internal reliability in the current study for the CSQ and the CDI; and adjustments for a range of potentially confounding variables that were collected by experienced psychology, physiology and cardiology staff. However, the current findings must also be interpreted in light of its limitations. Firstly, concerning the psychological assessment instruments, despite the care taken to maximize student understanding, the youngest children in particular may have had difficulty interpreting questionnaire items correctly. While other valid assessment of both stress and psychopathology were considered, including, clinical interviews and saliva assessed cortisol (although, this being a measure of stress response rather than the psychosocial experience of “distress” made it less appropriate in the LOOK Study), given the large scale of the current study, which involved repeated measures over a decade, this approach was not feasible. Self-report assessments are commonly used in children. Many self-report instruments of stress and depressive symptoms have been successfully implemented with younger populations (including the CSQ; [4]), are common and have demonstrated sound validity and reliability. In particular, it is worth noting that both the CSQ and CDI have been used to detect meaningful and significant relationships with a range of psychological (e.g. body image [51]) and physiological (e.g. insulin sensitivity and percent body fat [40]; physical activity and fitness [29]) indicators of health in other areas of the LOOK Study, providing good assurance of the methodological validity of these assessment tools in the current study. Secondly, while the EndoPAT system has emerged as a promising tool for investigating endothelial function among adults, this method is still relatively new to paediatric investigations [31], suggesting that ongoing research may lead to refinements in the system as a diagnostic instrument in children. In light of these limitations, despite using the best instruments available to measure stress, depression and endothelial function in a large cohort study, there is the potential that our currently available instrumentation are not sufficiently sensitive.

## Conclusion

In contrast to previous findings in youth with clinically diagnosed stress or depression, our data provided little evidence of any relationship between current or previous psychosocial stress or depression and endothelial function in 16-year-old boys and girls from the general population. However, our data need to be interpreted alongside the potential limitations in the sensitivity associated with currently available instrumentation for detecting psychological health and endothelial function of children. The research reported here may help guide future efforts in this area, highlighting the need for refinement in our current assessment tools in children. Despite these negative findings, this research is important in advancing our understanding of the origins and critical periods of associations between psychological health and vascular dysfunction, which may not emerge until late adolescence/early adulthood.
